# Frequency control of the islanded microgrid including energy storage using soft computing

**DOI:** 10.1038/s41598-022-24758-6

**Published:** 2022-11-27

**Authors:** Masoud Dashtdar, Aymen Flah, Seyed Mohammad Sadegh Hosseinimoghadam, Attia El-Fergany

**Affiliations:** 1 Department of Electrical Engineering, Islamic Azad University, Bushehr, Iran; 2 Processes, Energy, Environment, and Electrical Systems (Code: LR18ES34), National Engineering School of Gabès, University of Gabès, Gabès, Tunisia; 3 Electrical Engineering Department, Bushehr Branch, Islamic Azad University, Bushehr, Iran; 4 Electrical Power and Machines Department, Faculty of Engineering, Zagazig University, Zagazig, 44519 Egypt

**Keywords:** Energy science and technology, Engineering

## Abstract

Today, with the increasing penetration of microgrids, the degree of complexity and non-linearity of power systems has increased, causing conventional and inflexible controllers not to perform well in a wide range of operating points. In this paper, a self-tuning proportional-integral (PI)-controller based on a soft computation of a combination of genetic algorithm (GA) and artificial neural network (ANN). The GA-ANN is used to control the frequency of a microgrid in an island mode to automatically adjust and optimize the coefficients of a PI-controller. The proposed PI-controller is located in the frequency control secondary loop of an island microgrid. Since the ANN is a local search algorithm and can be located in local minimum points and on the other hand improving its performance requires a lot of training data. The ANN parameters are optimized using the GA algorithm's proposed controller. Train ANN online to adapt to the system and change the PI-control coefficients without a lot of training data, in addition to avoiding being in the local minimum points.The microgrid tested included various distributed generation units including battery energy storage that tried to create a more realistic frequency response for the microgrid by considering nonlinear factors on the model of these resources. Finally, the simulation results with different perturbations indicate the proper performance of the proposed controller.

## Introduction

Distributed generation (DG) is a source for producing electrical power with a capacity of less than 10 MW. It is frequently connected to distribution-side power systems and aids in power supply. For the purpose of power systems, the principal energy in these sources is clean and renewable energy from sources like wind, solar, and geothermal energy, which is utilized in the construction of wind turbines, solar cells, gas microturbines, fuel cells, etc.^1,2^. With the advent of DG, several problems appeared, including the maintenance and protection of resources. The issue relates to how these resources can help manage the grid's fundamental elements, such as frequency and voltage, and how electricity is transferred between the grid and DGs. The idea of microgrids was established in contemporary power systems to address these issues and take these resources and local demands into account in an integrated manner. This introduction defines microgrids as compact power grids made up of a number of DG sources and local loads. The microgrids are normally connected to the grid, but in case of an emergency brought on by the occurrence of severe disruptions, they are cut off and can provide the local loads on their own. When connected to the grid, the microgrid's frequency and power are functions of the main grid and only need to be controlled for the power of the units, but on islands, the microgrid's frequency and voltage fluctuate need an independent control^3,4^.

### Frequency control for microgirs in the litterature

Increasing the number of microgrids in power systems has changed the fundamental rules in these systems and caused the generation of resources to be distributed throughout the system. This causes the complexity and non-linearity of power networks to increase, and as a result, we do not see the proper response of conventional controllers as before. PI-controllers are most widely used in power systems because they have a simple structure and are cost-effective, and in power systems, these controllers are trusted more than any other controller. But the problem with these controllers is that the control coefficients based on the linear conditions and the operating point of the system are adjusted by the technicians based on their knowledge and experience, and are placed in the system at once. If the nominal operating conditions change or the linear conditions of the system change due to disturbances, the values intended for these controllers will no longer be optimal ​​and will not have the same proper response as before. The possible solution, both to use these conventional and reliable controllers and to somehow solve their problem, is to update and optimize the control coefficients depending on the changes in the system^5–7^.

Numerous references have reviewed and presented various methods for frequency control of microgrids based on the optimization of controller coefficients with meta-heuristic algorithms. In^8,9^, controllers based on PI control and proportional-integral-derivative controller (PID) have been used. In^10^ the particle swarm optimization (PSO) algorithm and in^9^ the spider social behavior (SSO) algorithm is used to optimize the PID control parameters in the microgrid. In^11^, the harmonic search (HS) algorithm is used to control the load–frequency in the microgrid. In^12^ uses a fuzzy controller whose coefficients are optimized using the PSO algorithm. In^13,14^ the model predictive control (MPC) is used to control the load–frequency of the microgrid. In^15^, a fuzzy controller is used to control the frequency of a multi-microgrid. In^16^ two-level MPC control^17^, multiple MPC control, and^18^ MPC control-based method for coordinated control of wind turbine blades and electric hybrid vehicles to reduce power fluctuations and microgrid frequency are presented. In^19^ the Ziegler-Nichols-based PID method (ZN-PID), in^20^ the fractional-order-based PID method (FOPID), in^21^ the fuzzy control based fractional order PID (Fuzzy FOPID), In^22^ the kriging based surrogate fractional order PID method has been used.

The methods proposed for the adaptive PI-controller are generally limited to linear processes. In other words, a controller with a linear model operates in a linear range, but due to the capabilities of ANN in solving problems with high mathematical complexity and the high power of these networks in estimating functions, designers are encouraged to use these networks in the design of self-tuning controllers to control nonlinear processes^23^. In^24–27^, a PI-controller with a hybrid ANN form is used as a direct adaptive controller to control the microgrid frequency, in which PSO and fuzzy algorithms are used to optimize ANN coefficients and their rapid training.

### Paper contribution

According to several studies done on the subject's state of the art, the typical PI-controller has not performed well due to the nonlinearity of the whole system. This is why, this paper presents an approach of a PI-controller self tuning. It is evident that a number of methods, including PSO and others, were suggested to aid in the automatic adjustment of the PI values. However, each of these approaches have their weakness and limitation, in relation the number of parameters that must be fixed at the beginning of the algorithm start. Therefore, this paper have merge two algorithms for making this automatic tuning touch and for compensate the weaknesses on the neural network offline learn. These two algorithms, compensate their limitations and present together a usuful solution for this Multi-input system. These two algorithms, which use the genetic algorithm approach (GA) and the neural network (NN) concept, respectively, have the advantages of training from the current state and optimal calculation specification, and this can be done online while the system is running, which makes it an advantage specification.This combination of the PI, NN and GA have given a best performances for this complex system stability in relation to the RMS (Δf) and max (|Δf|) of microgrid frequency changes..

### Paper structure

The following is the sections of the article. In “General microgrid structure and conventional control strategy” section, the microgrid structure with the conventional PI-controller is presented. “A proposed control strategy based on ANN-GA” section announces the proposed control strategy based on the combination of ANN and GA algorithms. In “Simulation results” section, the simulation results of the proposed method are exposed and discussed and finally, a conclusion will be presented in “Conclusions” section.

## General microgrid structure and conventional control strategy

Microgrids are a set of mainly renewable generators that are jointly formed to feed loads. The nature of microgrids is a wide-ranging distributed generation that itself has distributed generation resources. In a microgrid, we mainly deal with distributed generation sources such as solar cells, wind turbines (microturbines), fuel cells, batteries (energy storage systems), hybrid generators such as CHP, as well as synchronous generators. As it is known, the power output of these sources, except for synchronous and CHP generators, is DC. For this reason, we encounter two AC and DC links in a microgrid. This concept is called a hybrid microgrid. In addition to these sources, to benefit from the output power of each, control systems are needed, which are used to control the microgrid. The control strategies in microgrids should be such that they provide the basic purpose of these networks to continue to operate in both connection and disconnection from the main grid. For this purpose, two general control structures have been considered depending on the operating conditions of the microgrid. In case the microgrid is connected to the main grid, the stability of the basic parameters of the network such as voltage and frequency is provided by the main grid and the microgrid is considered an auxiliary element in providing common loads. This mode of operation is called PQ, which means that the microgrid is controlled for the delivery of fixed active and reactive power. In the event of a disconnection, the basic parameters of the system are set by the microgrid and it must otherwise supply its loads or at least critical loads. This mode of operation is called VSI. Therefore, to apply these control methods, a series of controllers are needed on each of the microgrid sources.

### Types of microgrid control

In a general sense, microgrid resources are divided into two parts: probabilistic and controllable generation. probability DG sources based on probabilistic (uncontrollable) inputs produce the desired output. These sources include solar cells, wind turbines, and even some fuel cells. These sources use sunlight, wind speed, and hydrogen, respectively, to generate power. Given that these inputs have probabilistic properties, these sources are also probability DG. Specifically, in controlling probability DG sources, we are faced with the problem of current control. In these sources, the output current (output power) of the system is controlled as CCS (controlled current source). But the important issue is the need for microgrid control over controllable resources. These sources can be such as the battery, CHP, or synchronous generator. What is required is the presence of at least one of these resources in a microgrid (in terms of microgrid stability and reliability). Controllable microgrid resources play an essential role in controlling microgrids and thus achieving microgrid stability in terms of voltage/frequency. In general, and in a specific definition, an unstable microgrid is a microgrid in which voltage/frequency collapse occurs. Voltage/frequency collapse in a microgrid means continuous increase or continuous decrease of the desired variable. On the other hand, in the microgrid, we also face the phenomenon of drop. At the opposite point of drop, there is also drop control. In the drop phenomenon, we encounter a voltage/frequency error (steady-state error).

The basis of stability in the microgrid was based on controllable resources. In these sources, the more accurate, robust, and practical the control process used, the more it improves the stability of the microgrid. For this purpose, different control levels are used sequentially in a microgrid. Each of these control levels is responsible for part of the microgrid stability tasks. In a microgrid, these levels are divided into three parts:

- Primary control level: In this control, the initial stability of frequency/frequency angle is considered. This type of control is responsible for preventing voltage/frequency collapse. One of the most common methods for this purpose is frequency drop control.
- Microgrid secondary control level: In this frequency/voltage drop control, the goal is stability. In the sense that events such as islanding or load change and even the occurrence of an error can cause a steady-state error in the underlying microgrid variables. This type of control is used for this purpose.

**Figure 1:**
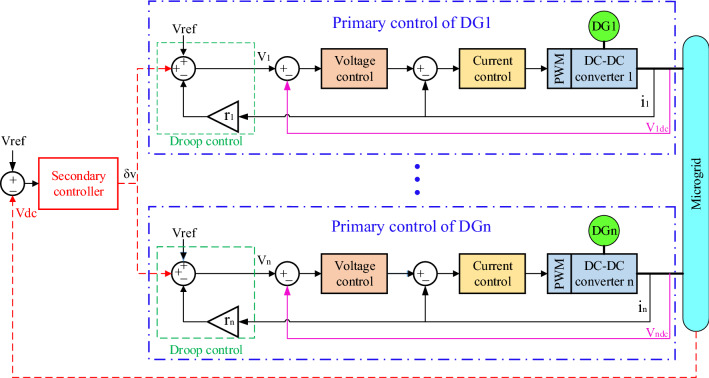
Primary and secondary control in the microgrid.

Figure 1 shows a view of the primary and secondary controls in the microgrid. In this control, the goal is to establish stability for voltage and frequency. In the presence of secondary control, this will also be the case when it becomes an island from the upstream network. This means that the frequency and voltage drop are compensated. But in addition to these two levels of control, optional controllers are also used. These controllers are responsible for improving the control process as much as possible. These controllers can be operated in parallel at any of the control levels. These types of controllers include fuzzy logic controllers, nonlinear, robust, adaptive controllers, etc. Each of these controllers according to their characteristics improve the microgrid status in terms of reliability, improving time characteristics (such as microgrid fluctuations), robustness to microgrid uncertainty, adaptation to variable parameters, etc. used.

**Figure 2:**
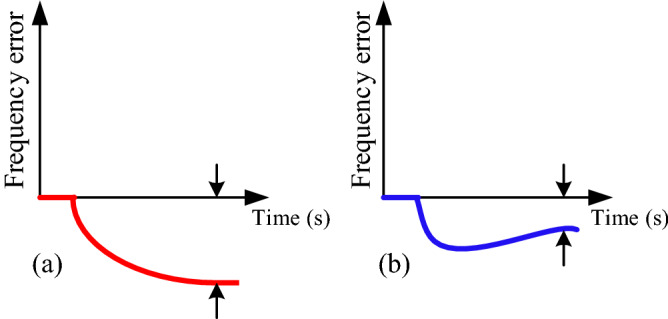
System frequency, (**a**) without a primary controller, (**b**) with a primary controller.

If there is a disturbance in the power system and it disturbs the balance between generation and consumption, the frequency will fluctuate. For example, if the load increases suddenly, the frequency will drop from the nominal value, which if not controlled and limited, will see frequency instability. Here, the primary control loop is the first control loop to limit the frequency drop after disturbance. Based on the frequency-active power characteristic of a generator, this control loop operates according to Eq. (1) and this loop is installed on the generator itself.

1 $$f-{f}_{0}=-{k}_{p}\left(P-{P}_{0}\right)$$

where *f*_0_ and P_0_ are the rated frequency and power of the network, respectively. The status of the frequency change in the presence and absence of the primary controller is shown in Fig. 2.

The primary control loop limits the dropped frequency but is unable to return the frequency to the nominal value hence the secondary control loop is used. In this control loop, conventional PI-controllers are often used to return the frequency to the initial value. Adjusting these controllers will be more based on classic methods and trial and error. The problems of these methods were mentioned in the introduction, and based on these reasons, in this article, while using these controllers, we have tried to solve their problems by using an intelligent method based on ANN.

### Structure of proportional-integral-derivative (PID) controllers

**Figure 3:**
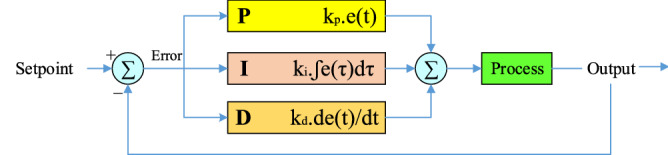
PID controller structure.

The PID-controller is a control system based on feedback, the main purpose of which is to bring the final result of the process closer to the desired value. The goal of a PID-controller is to steer the system toward a level, position, or whatever value we specify. According to the structure presented in Fig. 3, the two definitions "error" and "SetPoint" is of great importance in the PID-controller. Setpoint here means the target point (level, position, quantity, or whatever we want to reach in the control system) and on the other hand, the error is the amount of deviation (difference) between the target point and the final output value. Needless to say, the lower the error, the better, which means that we have been able to match the final value of the system exactly to our intended value. To reach this target point (error = zero, system output value = SetPoint), the PID control system uses three operators: Proportional, Integral, and Derivative. These three basic coefficients are variable in each PID-controller for specific applications to achieve the optimal response.

The three operators of the PID-controller, each of which receives the error signal as input and performs an operation on it, and finally their output is aggregated. The output of this set according to Eq. (2) is the same as the output of the PID-controller.

2 $$\begin{array}{c}output\left(t\right)= {k}_{p}e\left(t\right)+{k}_{i}\underset{o}{\overset{t}{\int }}e\left(\tau \right)d\tau +{k}_{d}\frac{de(t)}{dt}\\ {G}_{c}={k}_{p}+\frac{{k}_{i}}{s}+{k}_{d}s\end{array}$$

**Figure 4:**
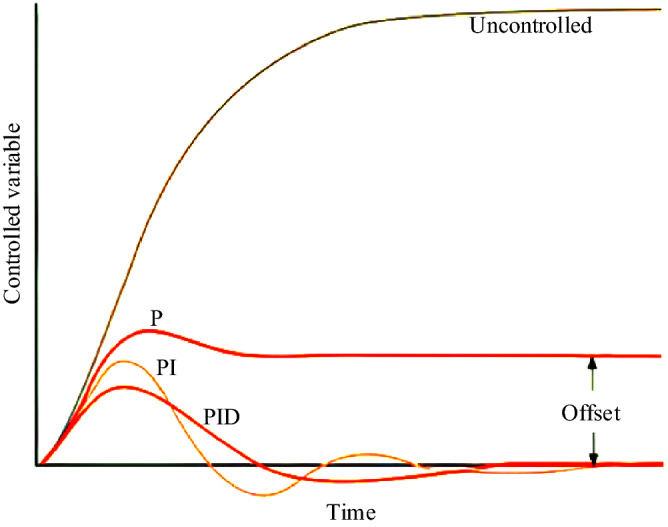
Comparison of three modes P, PI, and PID.

By combining three proportional-integral-derivative operators differently, we will have a different response to the error. The amount of response produced by each control mode can be optimized by changing its coefficient (k) and finally by combining these three main control modes to achieve an optimal PID system. Figure 4 compares the results of combining these three control modes (proportional-integral-derivative). P mode is usually used when the presence of offset in the system is not important and tolerable or when the process is naturally integral. PI mode is used when offset is not tolerable and there should be no steady-state error. PID is our choice when it is important to compensate for some natural inertia throughout the system and the process signals are relative without noise. According to the mentioned features, since the absence of steady-state error is important in controlling the microgrid frequency and the system is noisy, a PI-controller is usually used instead of a PID. Because the derivative mode of the PID-controller increases the effect of system noise and the performance of the controller will be different from the desired answer.

In a microgrid, the total generation power of units (P_GEN_) must be carefully controlled based on the load requirements so that a balance of generation power and consumption is established. The difference between the generated power and the load consumption can be expressed as Eq. (3).

3 $$\Delta P= {P}_{GEN}-{P}_{Load}$$

By controlling ΔP and Δf, the system can deliver good-quality power to the load. The frequency changes Δf can be calculated from the net power changes ΔP and are expressed in ideal conditions of Eq. (4):

4 $$\Delta f=\frac{\Delta P}{{K}_{sys}}$$

where K_sys_ is the constant frequency characteristic of the system. In real and practical terms, there is a time delay (T_sys_) in the frequency characteristic. Therefore, the function of converting system frequency changes to power changes (p.u.) is expressed as Eq. (5):

5 $${G}_{sys}=\frac{\Delta f}{\Delta P}=\frac{1}{{K}_{sys}\left(1+s{T}_{sys}\right)}=\frac{1}{D+Ms}$$

Here M and D are equivalent to the inertia and damping constants of the system, respectively. Frequency deviation is detected using the 1/D + Ms, which is characteristic of the system.

**Figure 5:**
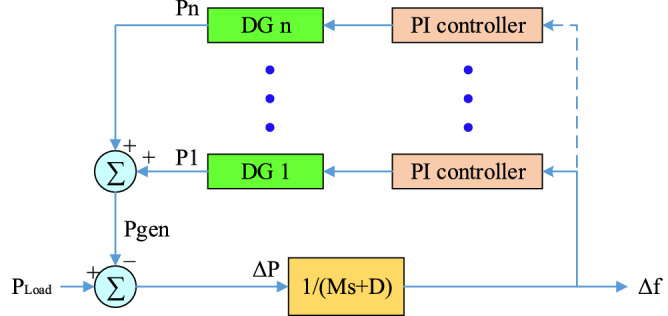
Microgrid frequency control based on PIcontroller.

According to Fig. 1, the block diagram of the frequency control method using the PI-controller can be shown in Fig. 5. Where proportional to the frequency deviation, each unit must change its output power so that the frequency deviation Δf has its lowest value. Determining the reference power of each unit is the responsibility of the integral controller, the output of which is determined based on the frequency deviation input.

### Dynamic modeling of microgrids under study

**Figure 6:**
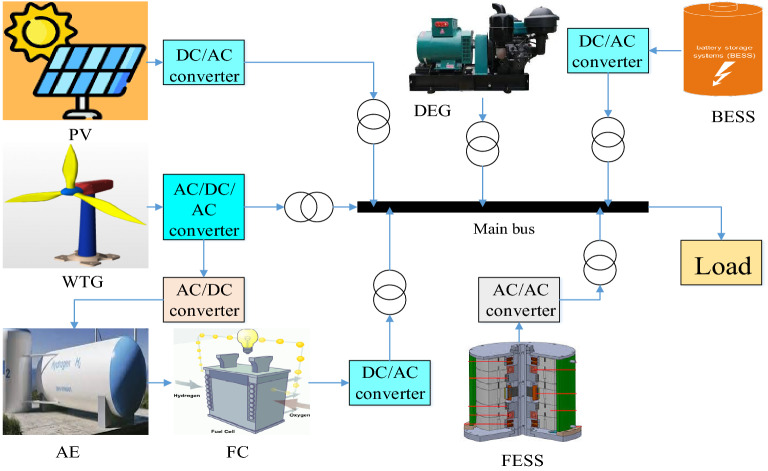
Microgrid under study.

**Figure 7:**
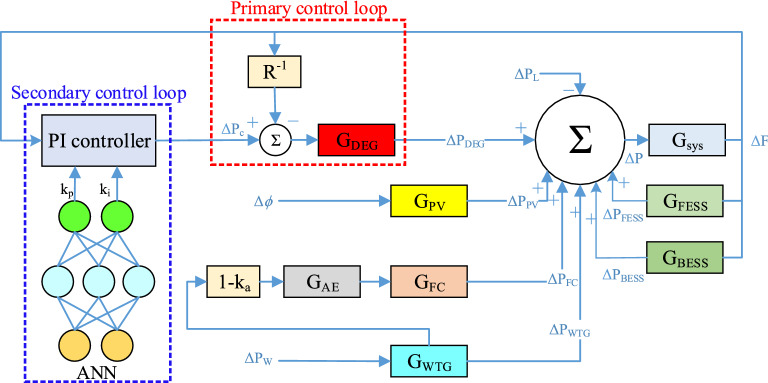
Microgrid frequency response model.

In this paper, a microgrid separate from the main grid is considered as the system under study, which is shown in Fig. 6. The microgrid consists of units including a diesel energy generator (DEG), a photovoltaic (PV), a wind turbine generator (WTG), a fuel cell (FC), an aqua electrolyzer (AE), a battery energy storage system (BESS), and a flywheel energy storage system (FESS). Given the focus of this paper on system frequency stability, a simplified model of the system frequency response is provided in Fig. 7 for a simpler analysis of how it behaves in the encounter of various disturbances. The values of the parameters used are presented in Table 1.

**Table 1:** The values of the parameters used in the microgrid frequency model.

Parameter	Value	Parameter	Value
D (pu/HZ)	0.012	T_WTG_ (s)	1.5
2H (pu.s)	0.1667	T_AE_ (s)	0.5
T_FESS_ (s)	0.1	K_WTG_	1
T_BESS_ (s)	0.1	R (HZ/pu)	3
T_FC_ (s)	4	K_a_	0.6
K_FC_	1/100	K_DEG_	1/300
K_AE_	1/500	K_FESS_	−1/100
K_BESS_	−1/300	K_PV_	1
T_DEG_ (s)	2	T_PV_	1.8

As you can see in Fig. 7, a PI-controller is designed to maintain microgrid stability, which is initially configured by the Ziegler-Nichols method, which is one of the strongest classical methods for adjusting control coefficients, and then optimized and configured online by the proposed method based on ANN. According to Fig. 7, the characteristic function of production units is expressed through Eq. (6) to Eq. (13). ^29^.

6 $$\begin{array}{c}{\Delta P}_{WTG}=\frac{{k}_{a}{k}_{WTG}{\Delta P}_{W}}{{T}_{WTG}}-\frac{{\Delta P}_{WTG}}{{T}_{WTG}}\\ {G}_{WTG}\left(s\right)=\frac{{k}_{a}{k}_{WTG}}{1+s{T}_{WTG}}=\frac{{\Delta P}_{WTG}(s)}{{\Delta P}_{W}(s)}\end{array}$$

7 $$\begin{array}{c}{\Delta P}_{PV}=\frac{{k}_{PV}\Delta \varnothing }{{T}_{PV}}-\frac{{\Delta P}_{PV}}{{T}_{PV}}\\ {G}_{PV}\left(s\right)=\frac{{k}_{PV}}{1+s{T}_{PV}}=\frac{{\Delta P}_{PV}(s)}{\Delta \varnothing (s)}\end{array}$$

8 $$\begin{array}{c}{\Delta P}_{DEG}=\frac{{k}_{DEG}{\Delta P}_{C}}{{T}_{DEG}}-\frac{{k}_{DEG}\Delta F}{R{T}_{DEG}}-\frac{{\Delta P}_{DEG}}{{T}_{DEG}}\\ \left\{\begin{array}{c}{G}_{DEG}\left(s\right)=\frac{{k}_{DEG}}{1+s{T}_{DEG}}=\frac{{\Delta P}_{DEG}(s)}{{\Delta U}_{DEG}(s)}\\ {\Delta U}_{DEG}\left(s\right)={\Delta P}_{C}\left(s\right)-\frac{\Delta F(s)}{R}\end{array}\right.\end{array}$$

9 $$\begin{array}{c}{\Delta P}_{AE}=\frac{{k}_{AE}\left(1-{k}_{a}\right){\Delta P}_{WTG}}{{T}_{AE}}-\frac{{\Delta P}_{AE}}{{T}_{AE}}\\ \left\{\begin{array}{c}{G}_{AE}\left(s\right)=\frac{{k}_{AE}}{1+s{T}_{AE}}=\frac{{\Delta P}_{AE}(s)}{{\Delta P}_{t}(s)}\\ {\Delta P}_{t}\left(s\right)=\left(1-{K}_{t}\right){\Delta P}_{WTG}\left(s\right), {K}_{t}=0.6\end{array}\right.\end{array}$$

10 $$\begin{array}{c}{\Delta P}_{FC}=\frac{{k}_{FC}{\Delta P}_{AE}}{{T}_{FC}}-\frac{{\Delta P}_{FC}}{{T}_{FC}}\\ {G}_{FC}\left(s\right)=\frac{{k}_{FC}}{1+s{T}_{FC}}=\frac{{\Delta P}_{FC}\left(s\right)}{{\Delta P}_{AE}\left(s\right)}\end{array}$$

11 $$\begin{array}{c}{\Delta P}_{\mathrm{BESS}}=\frac{{k}_{\mathrm{BESS}}{\Delta U}_{\mathrm{BESS}}}{{T}_{\mathrm{BESS}}}-\frac{{\Delta P}_{\mathrm{BESS}}}{{T}_{\mathrm{BESS}}}\\ {\Delta P}_{\mathrm{FESS}}=\frac{{k}_{\mathrm{FESS}}{\Delta U}_{\mathrm{FESS}}}{{T}_{\mathrm{FESS}}}-\frac{{\Delta P}_{\mathrm{FESS}}}{{T}_{\mathrm{FESS}}}\end{array}$$

12 $$\begin{array}{c}{G}_{\mathrm{BESS}}\left(s\right)=\frac{{k}_{\mathrm{BESS}}}{1+s{T}_{\mathrm{BESS}}}=\frac{{\Delta P}_{\mathrm{BESS}}\left(s\right)}{{\Delta F}_{\mathrm{BESS}}\left(s\right)}\\ {G}_{\mathrm{FESS}}\left(s\right)=\frac{{k}_{\mathrm{FESS}}}{1+s{T}_{\mathrm{FESS}}}=\frac{{\Delta P}_{\mathrm{FESS}}\left(s\right)}{{\Delta F}_{\mathrm{FESS}}\left(s\right)}\end{array}$$

13 $$\left\{\begin{array}{c}\Delta f\times \frac{1}{R}=\Delta P,\\ \Delta f=f-{f}_{0},\\ \Delta P=P-{P}_{0},\end{array}\right.$$

## A proposed control strategy based on ANN-GA

**Figure 8:**
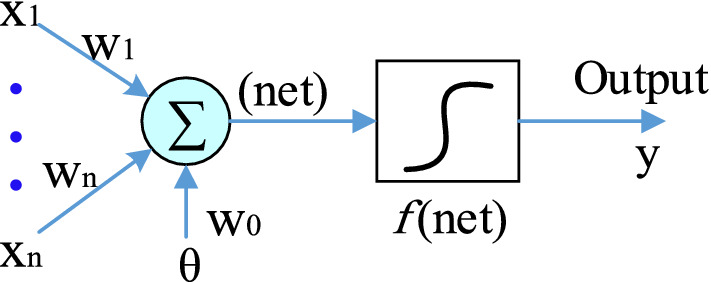
A simple mathematical model of neurons in ANN.

ANN is one of the most powerful tools in optimization processes because these networks have a wide ability to process and learn in parallel. Based on the structure of these networks and how the processing elements are combined, several important and basic applications such as mind modeling, financial modeling, time series prediction, control systems, and optimization for them are envisaged. To use ANN networks in the mentioned processes, it is necessary to consider a mathematical model of them. A simple mathematical model for analyzing their behavior is shown in Fig. 8. The vectors x, w, θ, and *f*(net) are input and weight vectors, bias values, and functions (linear or nonlinear) for neurons, respectively. The output of this model will be by Eq. (14).

14 $$y\left(k\right)=f\left(\sum_{j=1}^{n}{w}_{j}{x}_{j}\left(k\right)+{w}_{0}\theta \right)$$

**Figure 9:**
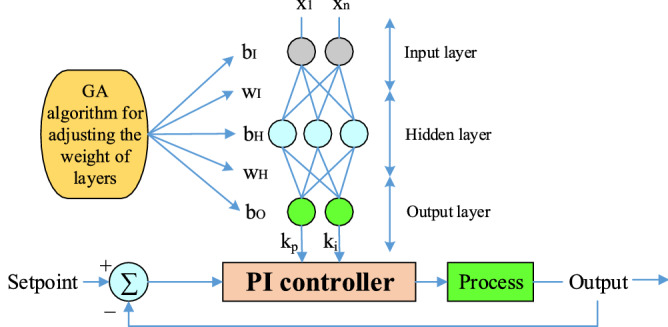
Proposed control structure for self-tuning PI-controller.

**Figure 10:**
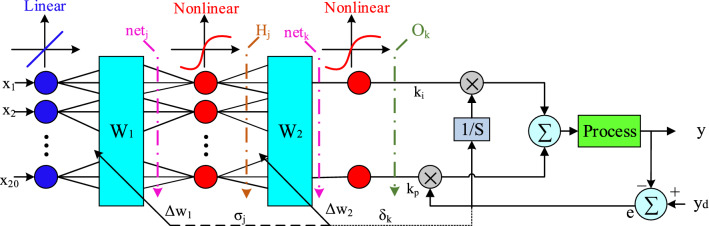
ANN structure for the online tuning of a PI-controller.

Figure 9 shows the control framework for the online tuning of a PI-controller used in the microgrid frequency secondary control process. To obtain the best performance of the PI-controller and determine the relevant coefficients, there are various practice methods. In this article, we have used the combined ANN-GA method to optimize PI coefficients. The structure for ANN to tune the PI-controller online is shown in Fig. 10. The considered network is a multilayer network in which 20 neurons are considered as the input layer (power changes and frequency deviations of units, etc.) and 2 neurons are considered as the output layer (according to the number of control coefficients to be adjusted). In Fig. 10 x, w_1_ and w_2_ are the input vectors and the weight vectors of the first and second layers, respectively. The functions considered in Fig. 10 will be linear for the first layer and nonlinear for the second and output layers. ANN first learns from training data how to change the coefficients to keep the system frequency constant, and then updates these coefficients optimally with the GA algorithm so that the controller is always set to the best values.

### Weight update based on error Back-Propagation algorithm

This section introduces the usual method for updating weights in ANN. The Back-Propagation method tries to have the minimum value of the performance function given in Eq. (15) in each weight update. y is the reference signal and y_d_ refers to the output of the output layers.

15 $$yE=0.5{\left(y-{y}_{d}\right)}^{2}$$

In this method, according to Fig. 10, the weights are updated to achieve the optimal values of the control coefficients (k_i_, k_p_) through Eq. (16). ^28^

16 $$\begin{array}{c}{w}_{2}\left(k+1\right)={w}_{2}\left(k\right)+{\Delta w}_{2}={w}_{2}\left(k\right)+\upeta \sigma H\\ {w}_{1}\left(k+1\right)={w}_{1}\left(k\right)+{\Delta w}_{1}={w}_{1}\left(k\right)+\upeta \delta x\end{array}$$

where Δw_1_ and Δw_2_ according to Eq. (17) and Eq. (18) are the vector of changes given in the initial values of the weights of the first and second layers so that the function is given in Eq. (15) to the smallest value, during the multi update. This operation tries to change the control coefficients so that the system frequency returns to its final value with the least fluctuation. All the parameters used in the calculation of Δw_1_ and Δw_2_, such as σ_j_, δ_k_, and H_j_, net_j_ can be seen in Fig. 10, and η = [0 1] was the learning rate.

17 $$\left\{\begin{array}{c}{\Delta w}_{2}=-\upeta \frac{\partial E}{\partial {w}_{2}}\\ \frac{\partial E}{\partial {w}_{2}}=\frac{\partial E}{\partial y}\cdot \frac{\partial y}{\partial u}\cdot \frac{\partial u}{\partial {net}_{k}}\cdot \frac{\partial {net}_{k}}{\partial {w}_{2}}\\ \begin{array}{c}\frac{\partial u}{\partial {net}_{k}}={f}^{^{\prime}}\left({net}_{k}\right),\frac{\partial {net}_{k}}{\partial {w}_{2}}={H}_{j}\\ \frac{\partial E}{\partial y}\cdot \frac{\partial y}{\partial u}\cdot \frac{\partial u}{\partial {net}_{k}}={\delta }_{k}\\ {\Delta w}_{2}=\upeta {\delta }_{k}{H}_{j}\end{array}\end{array}\right.$$

18 $$\left\{\begin{array}{c}{\Delta w}_{1}=-\upeta \frac{\partial E}{\partial {w}_{1}}\\ \frac{\partial E}{\partial {w}_{1}}=\frac{\partial E}{\partial y}\cdot \frac{\partial y}{\partial u}\cdot \frac{\partial u}{\partial {net}_{k}}\cdot \frac{\partial {net}_{k}}{\partial {H}_{j}}\cdot \frac{\partial {H}_{j}}{\partial {net}_{j}}\cdot \frac{\partial {net}_{j}}{\partial {w}_{1}}\\ \begin{array}{c}\frac{\partial u}{\partial {net}_{k}}={f}^{^{\prime}}\left({net}_{k}\right),\frac{\partial {net}_{k}}{\partial {H}_{j}}={w}_{2},\frac{\partial {H}_{j}}{\partial {net}_{j}}={f}^{^{\prime}}\left({net}_{j}\right) \\ \frac{\partial {net}_{j}}{\partial {w}_{j}}=x\\ {\Delta w}_{1}=\upeta {\delta }_{k}{f}^{^{\prime}}\left({net}_{k}\right){w}_{2}{f}^{^{\prime}}\left({net}_{j}\right)x=\upeta {\sigma }_{j}x\end{array}\end{array}\right.$$

### Weight update based on GA algorithm

One of the most important issues when implementing ANN is choosing the right training algorithm. The most common ANN training algorithm is the error back-propagation algorithm. The problem with this algorithm is slow convergence and stopping at optimal local points. One approach to ANN training is to use metaheuristic algorithms such as GA. In each cycle of this training, the weighting of the parameters is done by the GA algorithm. In the training method in this section, the GA algorithm first calculates the value of the cost function for the system response by selecting a random population as the ANN weights and changes the ANN weights accordingly to improve the ANN performance and minimize the value of the cost function. Here the process of this training method is called the self-modifying method. In this method, ANN weights are quantified as separate sections, each weight changes the ANN performance change, and the effect of each weight on ANN performance is determined, by using the intelligent GA algorithm, these changes are directed towards optimizing the ANN performance. Finally, the ANN weight coefficients are adjusted by optimizing the system efficiency. Therefore, in this method, there is no need to produce a lot of training data for ANN training, and by saving computational resources, higher accuracy can be achieved with less repetition.

The mathematical logic of the GA algorithm tries to optimize the output of the control system by minimizing an objective function. The aim here is to minimize Eq. (15) and considering that in this paper the goal is to improve the PI coefficients to control the microgrid frequency, two factors can be defined as the maximum overshoot (OS) rate and settling time (ST) of the frequency signal as Eq. (19)^30.^

19 $$\begin{array}{c}F=\alpha \cdot OS+(1-\alpha )\cdot ST\\ Assuming \alpha =0.5, F=0.5(OS+ST)\end{array}$$

By adding Eq. (19) to Eq. (15), a new objective function can be defined by Eq. (20) to improve the performance of the PI-controller in OS and ST control of microgrid frequency with better accuracy.

20 $$E=0.5{\left(y-{y}_{d}\right)}^{2}+F$$

**Figure 11:**
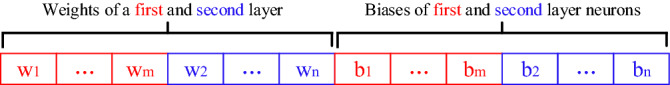
Structure of a chromosome for the proposed ANN training.

**Figure 12:**
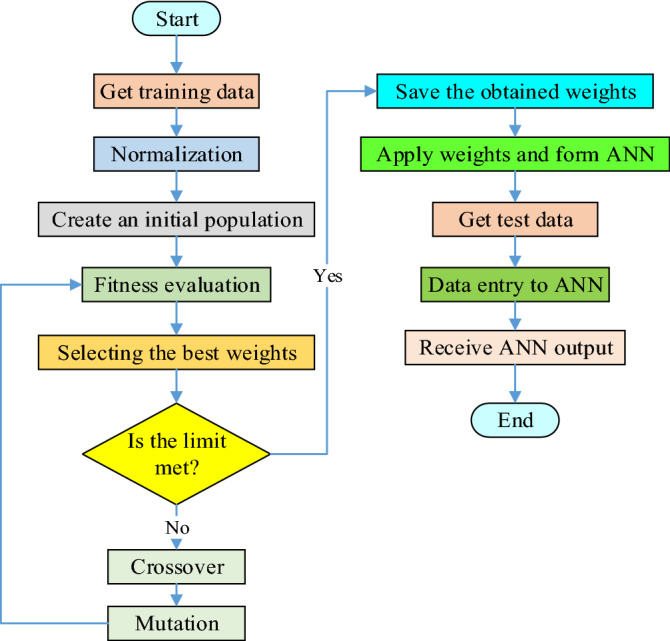
Flowchart of the proposed controller design.

In this study, the purpose of the GA algorithm is to determine the optimal biases and weights of ANN so that Eq. (20) is minimized. In evolutionary algorithms such as GA, the interface between the algorithm and the problem is how the chromosomes (solutions) are encoded and displayed. In the proposed GA algorithm, each chromosome represents the values of the weights and bias of the ANN network, so that the w_1_ to w_m_ genes represent the weights of the first layer and the w_2_ to w_n_ genes represent the weights of the second layer of ANN, and the genes b_1_ to b_m_ and b_2_ to b_n_ represent the bias values of first and second layer neurons. Each gene can produce a real value in the range of -1 to 1. Figure 11 shows the structure of a chromosome proposed for ANN training. Finally, Fig. 12 shows the whole design process of the PI-controller from the combination of ANN and GA algorithms as a flowchart.

## Simulation results

**Figure 13:**
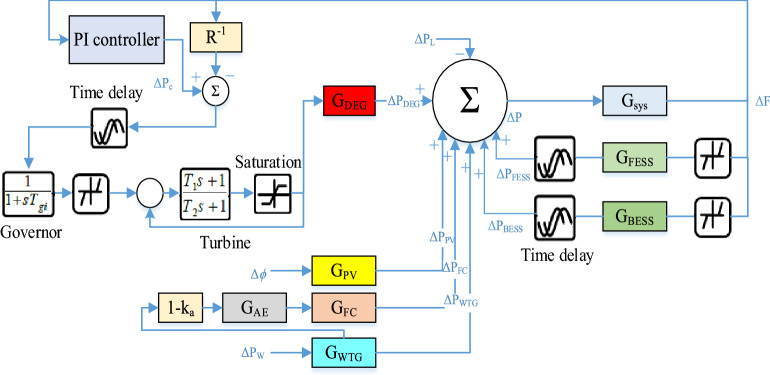
Microgrid frequency model considering physical constraints.

As you know, various factors such as load changes, uncertainty, units power, nonlinear elements, noise, etc. have a direct impact on the microgrid frequency. In this section, to evaluate the performance of the proposed control method, several different disturbances are applied to the studied microgrid through MATLAB software and the performance results of the proposed method are compared with the conventional PI-controller. To improve the model and get closer to the actual microgrid response, a series of nonlinear elements, limiters, and time delays are added to the original frequency model, which is shown in Fig. 13. One of the most important physical limitations is related to the diesel generator, which due to mechanical and thermal limitations, is not able to respond to disturbances at the same time and there is always a delay between the occurrence of disturbances and the response to it. Also, due to the existence of different filters and telecommunication channels, there is a delay in transferring the measured parameters to the control systems. Therefore, due to the mentioned reasons, delay blocks have been added to the system model. For delayed cases, a time delay of one cycle (20 ms) is provided. Control signals can also be increased or decreased to a certain extent, and the production sources have a dead band that will not be activated until the control input signal to these sources reaches a certain level. The rate of increase or decrease in generator output is also limited. Therefore, according to these cases, the model intended for the diesel generator has been made more accurate by adding a non-linear block, according to what is shown in Fig. 13.

**Figure 14:**
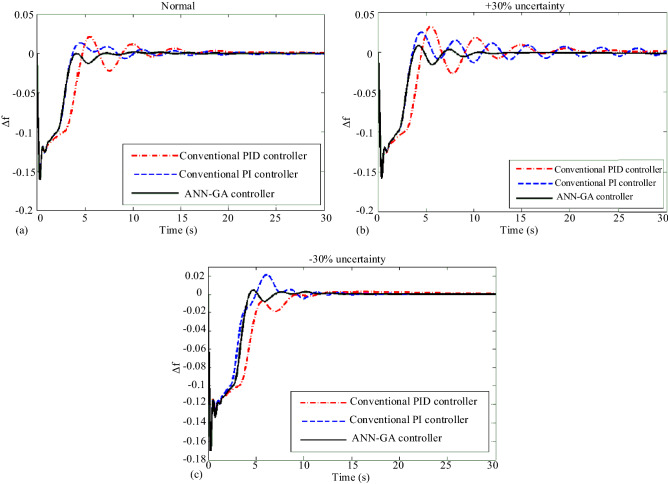
Scenario 1- Microgrid frequency response to step load perturbation for (**a**) normal state, (**b**) with + 30% uncertainty, (**c**) with -30% uncertainty.

In Scenario 1, a step overload of 0.1 pu is applied to the microgrid. Simulation for normal states and worst-case uncertainty in parameters, ie uncertainty ± 30% of the nominal value, are considered and compared with the performance of conventional PID and PI-controllers and the proposed intelligent controller, the results of which are shown in Fig. 14. The presence of nonlinear factors in the model of some microgrid components causes the microgrid structure to become more complex and the PID-controller to control frequency fluctuations does not perform as well as the PI-controller. However, the proposed controller, due to the nonlinear behavior of the microgrid at any time, applies the appropriate correction coefficients to the PI-controller and causes the PI coefficients to be adjusted adaptively and to control the fluctuations of the microgrid frequency well. In the simulation results of this scenario, it is observed that the proposed controller has significantly reduced the maximum overshoot and settling time of the microgrid frequency, especially when the system has uncertainty.

**Figure 15:**
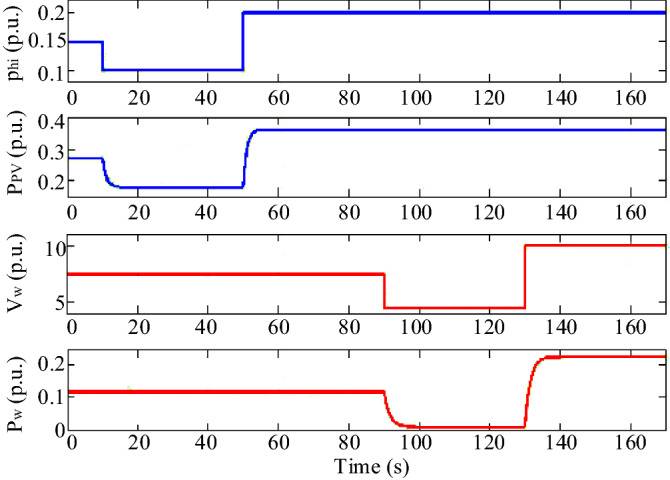
Scenario 2- Disturbances applied to wind speed and solar irradiation and related output powers.

**Figure 16:**
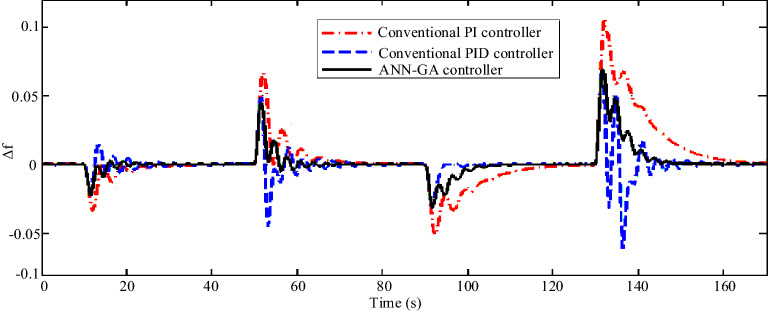
Scenario 2- The microgrid frequency response to changes in wind speed and solar irradiation.

In Scenario 2, a perturbation according to Fig. 15 is applied to the wind speed and solar irradiation, which also shows the power changes of the respective units. The perturbation of the solar irradiation is such that at t = 10 s the solar irradiation decreases from the initial value of 0.15 pu to 0.1 pu and increases at t = 50 s to the value of 0.2 pu. The perturbation at the wind speed is such that at t = 90 s, the wind speed decreases from 7.5 m/s to 4.5 m/s and increases to 10 m/s at t = 130 s. The microgrid frequency response by applying these perturbations is shown in Fig. 16. In this scenario, the superiority of the proposed controller performance over PID and PI-controllers in damping microgrid frequency fluctuations is well observed.

**Figure 17:**
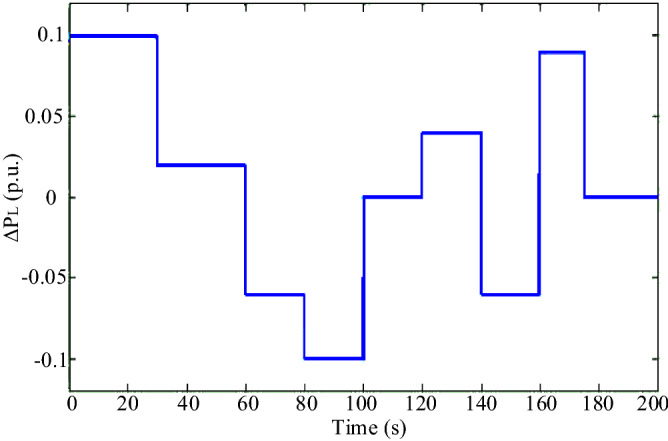
Scenario 3- Load perturbation applied to the microgrid as an irregular pulse train.

**Figure 18:**
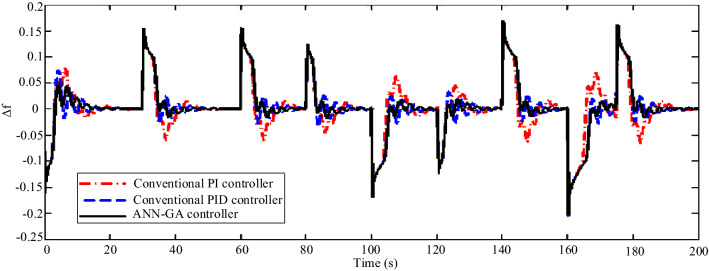
Scenario 3- Microgrid frequency response to an irregular pulse train.

In Scenario 3, to model instantaneous load changes, the load perturbation is applied to the microgrid in the form of an irregular pulse train shown in Fig. 17. The performance of the proposed controller and how to track the load is shown in Fig. 18. Load changes are always noticed by the microgrids and the microgrid controller must be able to quickly dampen the frequency fluctuations caused by the imbalance of production and power consumption in the shortest possible time and with the least fluctuations. In this scenario, the superiority of the ANN controller over conventional PID and PI can be seen. Using the proposed controller, after each load change, the system frequency changes return to normal with the least fluctuation and in the shortest settling time, while with other controllers, the frequency has more fluctuations and returns to normal later.

**Figure 19:**
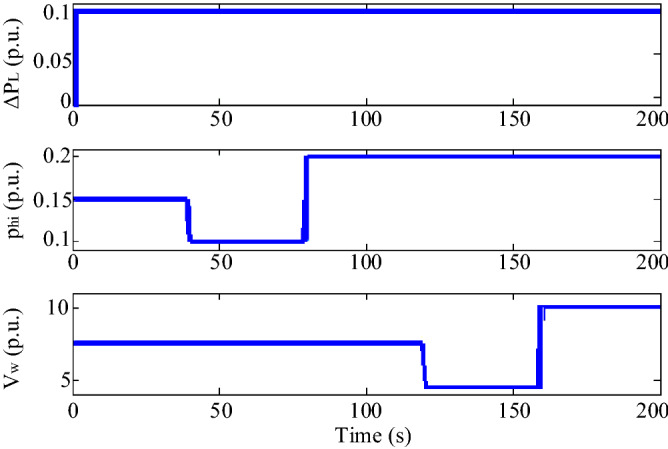
Scenario 4- Disturbances applied to solar irradiation, wind speed, and grid load.

**Figure 20:**
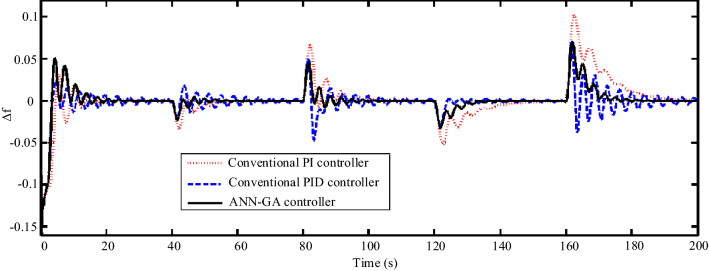
Scenario 4- Microgrid frequency response to solar irradiation, wind speed, and grid load under + 30% uncertainty.

In Scenario 4, the perturbations of the first and second scenarios are applied simultaneously. Also, to test the robustness of the controller in the worst case, perturbations are applied under + 30% uncertainty. The perturbations according to Fig. 19 are applied to the wind speed and solar irradiation as well as the load of the step. As shown in Fig. 20, the microgrid frequency response under these conditions is very favorable and has a relative advantage over other controllers.

**Figure 21:**
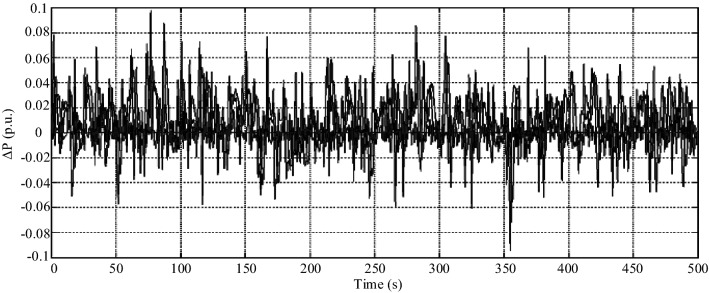
Scenario 5- White noise.

**Figure 22:**
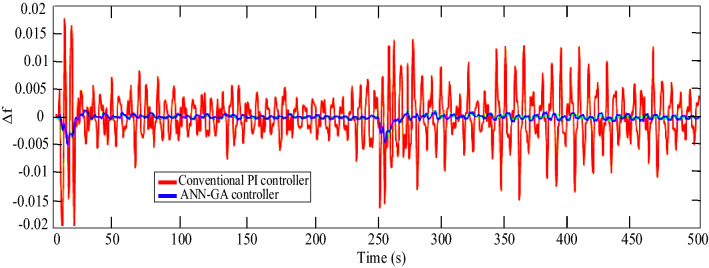
Scenario 5- Microgrid frequency response to white noise.

In scenario 5, to finally test the stability of the system with the proposed controller and show its better performance, white noise according to Fig. 21 is applied to this model and the microgrid frequency status in both modes of use of the proposed controller and the conventional PI-controller is shown in Fig. 22. As can be seen in this case, the designed intelligent controller has a much better performance.

In this section, the performance of the ANN-GA controller was evaluated in different scenarios and the results showed its superiority over conventional controllers in controlling the microgrid frequency under various disturbances. So far, most of the results shown were related to the performance of the proposed controller, the following results will be related to the performance of the artificial neural network itself will be presented. As you can see, most of the disturbances in the microgrid will involve the same five scenarios presented in this paper with some changes in production power, load changes, etc. Therefore, to evaluate the accuracy of the ANN proposed in this paper, the total data considered for evaluation was 205 cases, which were considered separately for each scenario according to Table 2.

**Table 2:** Total data generated.

Scenarios	Number of data
Scenario 1 (load range(pu) × uncertainty)	10 × 4 = 40
Scenario 2 (power range(pu) + time of disturbances)	24 + 24 = 48
Scenario 3 (Load range(pu) × time of disturbances)	10 × 5 = 50
Scenario 4	10 + 48 = 58
Scenario 5 (Noise on output power)	9
Total	205

**Figure 23:**
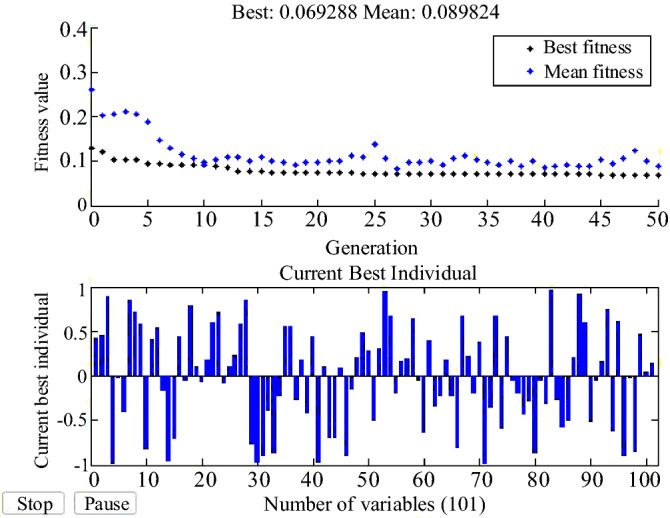
ANN training process by GA algorithm.

**Figure 24:**
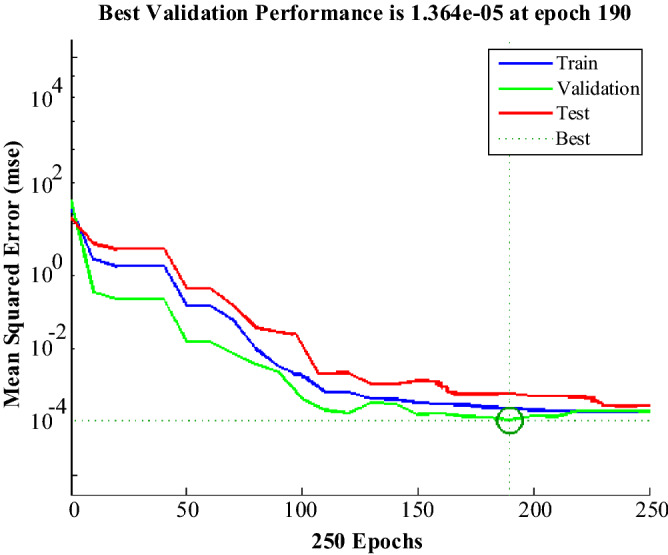
Performance diagram of the ANN.

According to the data generated in Table 2, the ANN network is trained by the GA algorithm. Figure 23 shows the performance of the GA algorithm in optimizing ANN weights and biases. In MATLAB, there are various diagrams to examine and show the performance of ANN networks. Figure 24 shows the ANN performance diagram. This diagram shows the number of iterations on the one hand and the mean square error (mse) of the ANN on the other hand, which in this design has reached 1.364e-05 in 190 iterations.

**Figure 25:**
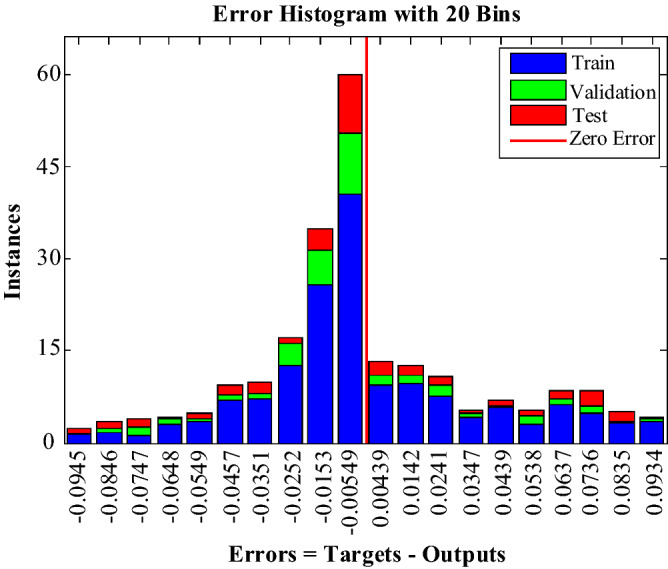
Error histogram diagram of the ANN.

Figure 25 shows the error histogram diagram for the ANN network. In this diagram, the degree of belonging of each data category for different errors is examined.

**Figure 26:**
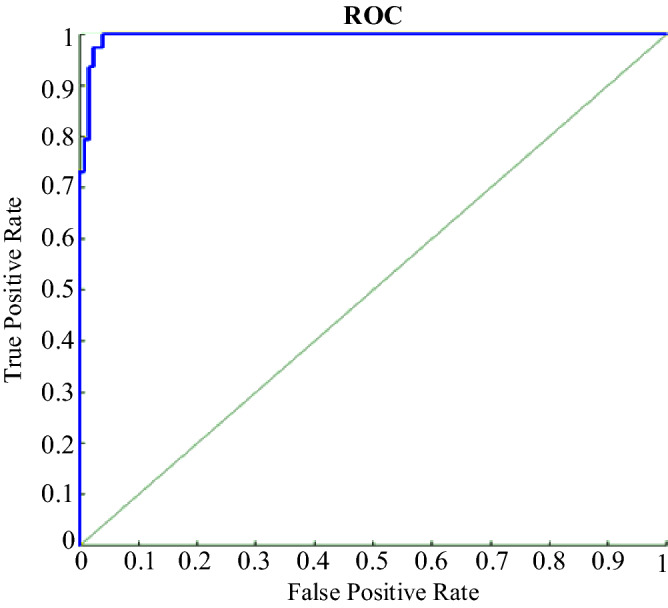
ROC diagram of the ANN.

Figure 26 shows the ROC diagram of the ANN network. In this diagram, the closer the points are to the top and left, the more appropriate it is, and the closer the forecast model is to its ideal state. The coordinates of the point (0,1) are ideal states. This point indicates that what the forecast model offers is fully consistent with the actual model. The opposite point has coordinates (1,0), which means that whatever prediction model is presented is the opposite of the actual model.

**Figure 27:**
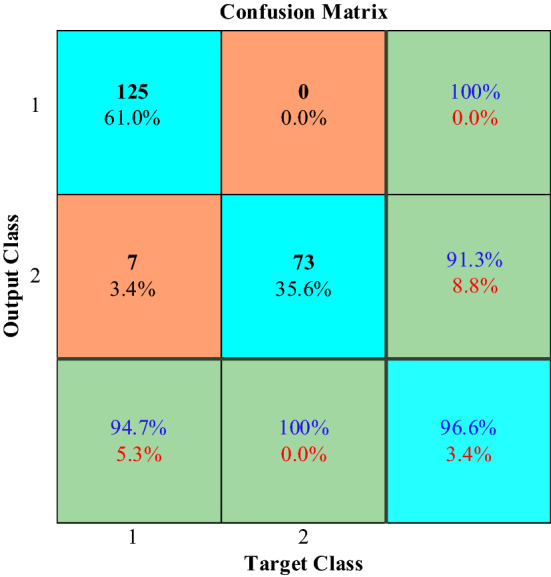
Confusion matrix of the ANN.

Figure 27 shows the confusion matrix of the ANN network. In this matrix, diagonal cells are related to correctly classified observations and non-diagonal cells are related to incorrectly classified observations with a percentage of performance. Here, the overall accuracy of the ANN network is 96.6%.

Finally, in scenarios, to implement the proposed controller adjusted with ANN-GA, the system is first set in the desired zero states, and then by introducing disturbances to the system, ANN-GA adjusts the parameters of the proposed controller so that it can move the system towards the optimal response. The optimal coefficients for this system obtained by ANN-GA are presented in Table 3. By comparing the coefficients obtained with the ANN-GA method and the coefficients of the Ziegler-Nichols method, it can be seen that the ANN-GA has been able to accurately perform this operation without the need for any predetermined data, and by choosing the correct control coefficients, the response of the system is towards the output to deliver the desired.

**Table 3:** Optimal coefficients of the PI-controller.

Methods	PI-controller coefficients	
	K_p_	K_i_
Ziegler–nichols	5	22
ANN-GA	4.09	20.42

A quantitative comparison of the performance of the proposed control method has been made with two indicators: RMS (Δf) (root mean square of frequency changes) and max (|Δf|) (maximum overshoot and undershoot). The improvement percentage of these two indicators in scenario 5 for a conventional PI-controller, conventional PID-controller, and ANN-GA method are shown in Table 4. As it can be seen, the proposed method has performed better.

**Table 4:** Percentage improvement of RMS (Δf) and max (|Δf|) indexes.

Methods	Indexes	
	RMS (Δf) (%)	Max (|Δf|) (%)
Conventional PI-controller	17	15
Conventional PID-controller	32	34
ANN-GA	46	47

## Conclusions

The balance between the production and consumption of active power is the main factor in ensuring the frequency stability of the microgrid. In this paper, an ANN-based PI-controller is proposed to control the microgrid frequency in the island mode. The proposed PI-controller structure is such that its coefficients are adjusted by ANN at any time according to system frequency changes. Since ANN design for frequency control required a lot of training data on ANN training, in this study, the GA algorithm was used to set and train ANN. The performance of the proposed controller was such that it could perform well for various types of disturbances under different scenarios and could be easily implemented due to the nonlinear and complex structure of microgrids. Also, to increase the efficiency of the controller in different operating points, the controller was designed by considering uncertainties in some microgrid parameters so that the proposed controller is robust to changes in the working points. Finally, the performance of the proposed controller was compared with conventional PI- and PID-controllers for different scenarios, which showed the appropriate accuracy of the proposed controller.

Even the good obtained results, this approach have some weaknesses and limitations. Basiacly, each control approach which is based on a neural network algorithm, will need a large database for having an optimal comoprtment. This is can be of the weaknesses of this part; it is hard for a standard calculator to manage a mega information. So, this is will force using a high resolution processor, which can be defficult in some positions and which increase the cost of the global control loop. In this same way, the integration of a two complex optimization algorithm, will make decision calculation hard and maybe can cause some delays on the system. Actually, this risk is low, but it can be happen in such cases. This caused delay, can make a risk to not detect a micro variation and not having the optimal decision on time.

From the other side, this work, have a potential ti be extended and ameliorated by testing more optimization solution, for testing the global comportment. Sliding mode control loop can be designed for controlling the frequency perturation and verify if it is more easy and rapid to have a stable performances after the coming perturbation on the grid side or from the different sources types. Also, testing this approch on a practical case, can be interesting to have a real result on this proposed approach. Maybe, it is better to test the execution rapidity of this control loop on this complex system and see if it is able to apply it really or not.

### Human and animal rights

This article does not contain any studies with animals performed by any of the authors.

## Data Availability

The data that support the findings of this study are available from the corresponding author upon reasonable request.
